# Alteration of serotonin transporter density and activity in fibromyalgia

**DOI:** 10.1186/ar1982

**Published:** 2006-06-21

**Authors:** Laura Bazzichi, Gino Giannaccini, Laura Betti, Giovanni Mascia, Laura Fabbrini, Paola Italiani, Francesca De Feo, Tiziana Giuliano, Camillo Giacomelli, Alessandra Rossi, Antonio Lucacchini, Stefano Bombardieri

**Affiliations:** 1 Department of Internal Medicine, Division of Rheumatology, University of Pisa, Via Roma 67 - 56126 PISA Italy; 2 Department of Psychiatry, Neurobiology, Pharmacology and Biotechnology, University of Pisa, Via Bonanno 6, 56126, Pisa, Italy

## Abstract

The aim of the study was to evaluate the kinetic parameters of a specific serotonin transporter (SERT) and serotonin uptake in a mentally healthy subset of patients with fibromyalgia. Platelets were obtained from 40 patients and 38 healthy controls. SERT expression and functionality were evaluated through the measurement of [^3^H]paroxetine binding and the [^3^H]serotonin uptake itself. The values of maximal membrane binding capacity (*B*_max_) were statistically lower in the patients than in the healthy volunteers, whereas the dissociation constant (*K*_d_) did not show any statistically significant variations. Moreover, a decrease in the maximal uptake rate of SERT (*V*_max_) was demonstrated in the platelets of patients, whereas the Michaelis constant (*K*_m_) did not show any statistically significant variations. Symptom severity score (tiredness, tender points index and Fibromyalgia Impact Questionnaire) were negatively correlated with *B*_max _and with *V*_max_, and positively correlated with *K*_m_. A change in SERT seems to occur in fibromyalgic patients, and it seems to be related to the severity of fibromyalgic symptoms.

## Introduction

Fibromyalgia syndrome (FMs) is a chronic pain syndrome characterized by widespread pain and stiffness, multiple tender points, and fatigue [1]. This pain syndrome has an incidence of 2% in the general population and occurs with higher frequency among women in middle age [2]. FM is often associated with increased prevalence of depressive symptoms, major depression and anxiety [3]. The cause and pathophysiology of FMs is unclear; pathophysiological hypotheses include impairment in the functioning of the hypothalamic-pituitary axis and alterations in specific neurotransmitters such as substance P, *N*-methyl-D-aspartate, noradrenaline (norepinephrine) and serotonin (5-HT). However, interest has been growing in a possible involvement of 5-HT in FM. Indeed, strong evidence has accumulated to support the hypothesis that a deficiency in serotonergic neuronal functioning might be related to the pathophysiology of FM [5-8]. Patients with FM have been found to have decreased concentrations of 5-HT and tryptophan (5-HT precursor) in serum and cerebrospinal fluid. 5-HT, in particular, is theorized to have a function in stage 4 sleep and in the pain threshold [9]. This neurotransmitter is implicated in psychiatric disorders such as depression, anxiety, and obsessive compulsive disorder. Stressful experiences lead to depression in some people who are already genetically predisposed, and increase the probability of FM exordium. The 5-HT gene could moderate the serotonergic response to stress [4].

As a mediator, 5-HT exerts its actions by means of interaction with distinct receptors, which are differentiated on the basis of structures, molecular mechanisms and pharmacological profiles [9]. On this basis, drugs acting on 5-HT receptors, in particular on 5-HT_2 _and 5-HT_3_, are being used or investigated for the clinical management of FM [10,11]. Among drugs targeting 5-HT receptors, ketanserin is a selective 5-HT_2 _antagonist that can reduce the hyperalgesia, spontaneous pain, sleep disorders and other symptoms of FM [12], and granisetron and tropisetron are selective 5-HT_3 _receptor antagonists that show clinical efficacy in FM [11].

In an analogous manner to other transmitters, the endogenous activity of 5-HT is controlled by a specific 5-HT transporter (SERT), which mediates the intracellular reuptake of 5-HT and can be specifically blocked by selective 5-HT reuptake inhibitors (SSRIs) such as paroxetine and fluoxetine. SERT is widely expressed in intestinal epithelial cells, in central or peripheral serotonergic neurons and in platelets; it shares common molecular and physiological features in these locations [13-15]. Clinical studies have demonstrated the efficacy of SSRI in FM [16], but the data are not unequivocal. Nociception refers to the physiological process of transmitting a painful stimulus from the periphery through afferent neurons to the cerebral cortex. It has been postulated that serotonergic neurotransmission has a significant function in nociception [17,18]; alterations in 5-HT metabolism and transmission might therefore be important in the pathogenesis of FM. These findings support the proposal that aberrant pain perception in FM also results from an instability of the 5-HT system in FM. There is also evidence that changes in the expression of SERT are due to a polymorphism in the transcription region in patients with FM [19].

In the present study, both the expression and functionality of SERT were determined in platelets collected from patients with FM, with the following aims: first, to perform a comparison with the pharmacological profile of platelet SERT in healthy volunteers, and second, to examine putative correlations of SERT characteristics with the severity of symptoms such as tiredness, Fibromyalgia Impact Questionnaire (FIQ) score or tender point index (TPi).

## Materials and methods

### Materials

[^3^H]Serotonin (specific radioactivity 30 Ci/mmol) and [^3^H]paroxetine (specific radioactivity 19.1 Ci/mmol) were purchased from Perkin-Elmer Life Science (Milano, Italy). All other reagents were obtained from normal commercial sources.

### Subjects

Forty patients (all female) affected by primary FM, aged 53 ± 13 years (mean ± SD) took part in the study, and 38 healthy females age-matched to the patients (50 ± 12 years) were used as a control group. The American College of Rheumatology criteria for FM [1] were used to make the diagnosis of FM. The inclusion criteria for the study groups comprised a negative history for psychoactive drug treatment and other neurological disorders. None of the subjects had comorbid psychiatric disorders or had received treatment with antidepressant drugs. No patient was under pharmacological treatment. All patients underwent a wash-out period of 2 months before the study. They were enrolled at the University Division of Rheumatology, Santa Chiara Hospital, Pisa. Written consent was obtained from all patients and controls after a full explanation of the procedure. The study was approved by the local Ethics Committee.

### Evaluation of clinical parameters

For each patient and control, tenderness at tender points was evaluated by means of the Fischer dolorimeter [20]. The total fibromyalgic tender point score (right plus left) was used for statistical analysis. Each positive tender point had a pain score between 0 (no pain) and 3 (severe pain). We also calculated the TPi as the sum of the scores of all tender points divided by the total number of tender points.

To estimate the impact of FM on the quality of life, all patients and controls received an FIQ [21]. The total score was the sum of the values of the 10 FIQ items, which reflected the impact of FM and ranged from 0 (no impact) to 100 (maximum impact).

Tiredness was evaluated as measured by the FIQ tiredness item, which consisted of a visual analogic scale numbered between 0 and 10.

To exclude major psychiatric disorders, all patients were evaluated by means of a diagnostic interview consisting of the administration of the Structured Clinical Interview for DSM-IV axis-I disorder (SCID-I/P) [22]. This assessment was conducted by psychiatrists who were trained and certified in the use of the study instruments at our department.

### Separation of platelets

Venous blood (30 ml) was collected from each subject and gently mixed with 1 ml of anticoagulant (0.15 M EDTA). Platelet-rich plasma was obtained by low-speed centrifugation (200*g *for 20 minutes at 22°C). Platelets were counted automatically with a flux cytometer (Cell-dyn 3500 system; Abbott, Milano, Italy).

For measurement of [^3^H]serotonin reuptake, platelets were used immediately, whereas for [^3^H]paroxetine binding, platelets were precipitated by centrifugation at 10,000*g *for 10 minutes at 4°C and the pellets were then stored at -80°C until the assay.

### [^3^H]Serotonin uptake

[^3^H]Serotonin uptake was performed as described by Arora and Meltzer [23], with some modifications. Aliquots of platelets (2 × 10^6 ^platelets) were incubated for 10 minutes at 37°C with [^3^H]serotonin at six concentrations from 15 to 700 nM in an assay buffer (118 mM NaCl, 4.7 mM KCl, 1.07 mM MgSO_4_.7H_2_O, 1.17 mM KH_2_PO_4_, 25 mM NaHCO_3_, 11.6 mM glucose, pH 7.4, in the presence of 0.1% ascorbate and 100 μM Pargyline) to a final volume of 0.5 ml. Non-specific uptake was measured in the presence of 10 μM Fluoxetine.

### Preparation of platelet membranes

The platelet pellets were washed with 10 ml of buffer (150 mM NaCl, 20 mM EDTA, 50 mM Tris-HCl, pH 7.4). Pellets were lysed and homogenized in 10 ml of buffer (5 mM Tris-HCl, 5 mM EDTA, pH 7.4, containing the following protease inhibitors: 200 μg/ml bacitracin, 160 μg/ml benzamidine and 20 μg/ml soybean trypsin inhibitor) with an Ultra-Turrax homogenizer and centrifuged twice at 30,000*g *for 15 minutes at 4°C. The resulting pellets were resuspended in ice-cold 50 mM Tris-HCl buffer, pH 7.4, containing protease inhibitors, and centrifuged at 50,000*g *for 15 minutes at 4°C. The pellets were then suspended with assay buffer (50 mM Tris-HCl, 120 mM NaCl, 5 mM KCl, pH 7.4).

Protein concentration was determined with the method of Lowry and colleagues [24] using bovine serum albumin as a standard.

### [^3^H]Paroxetine binding assay to platelet membranes

[^3^H]Paroxetine binding was performed as described by Marazziti and colleagues [25]. The incubation mixture consisted of 100 μl of platelet membranes (50 to 100 μg of protein per tube), 50 μl of [^3^H]paroxetine at six concentrations (0.01, 0.025, 0.05, 0.25, 0.5 and 1 nM) and 1.85 ml of assay buffer. Specific binding was obtained by using 10 μM fluoxetine as a displacer.

### Data analysis

Equilibrium-saturation binding data, the maximum binding capacity (*B*_max_, fmol/mg of protein) and the dissociation constant (*K*_d_, nM) were analysed by means of the iterative curve-fitting computer programs EBDA and LIGAND (Kell for Windows, v. 6.0).

The maximal uptake rate of SERT (*V*_max_, pmol/10^9 ^cells per minute) and the Michaelis constant (*K*_m_, nM) were obtained by direct weighted nonlinear regression of uptake rate against [^3^H]serotonin concentration with GraphPad PRISM software (GraphPad, San Diego, CA, USA).

Statistical analysis was performed with Student's *t *test. The relationship between variables was checked with a two-tailed Spearman analysis.

## Results

[^3^H]Paroxetine binding sites on the SERT of platelet membranes were used as a first marker of serotonergic function. Table 1 shows the mean *B*_max _values for the density, and *K*_d _values for the affinity, of [^3^H]paroxetine binding on platelets in the patients and in the healthy controls. A statistically significant difference was found between the two groups for *B*_max _(1,048 ± 30 versus 1,260 ± 34 fmol/mg for control; mean ± SEM) but not for *K*_d _(0.086 ± 0.021 nM versus 0.077 ± 0.012 nM for control; mean ± S.E.M.).

The evaluation of symptom severity in patients with FM (Table 2) yielded tiredness scores from 2 to 10 with a mean of 7.4 ± 2.2 (SD), TPi values from 1 to 3 with a mean of 2.2 ± 0.5 (SD), and FIQ scores from 8 to 94 with a mean of 57.1 ± 21.3 (SD). The severity of symptoms was shown to be related to the maximal binding capacity of platelet SERT, in that the statistical analysis showed a negative correlation between symptom scores and the respective *B*_max _values (*p *< 0.0001 for tiredness; *p *< 0.0001 for TPi and *p *< 0.0001 for FIQ). By contrast, no significant correlation was found when comparing symptom severity with *K*_d _values.

Age did not seem to influence the binding parameters of platelet SERT in either patients with FM or healthy volunteers. [^3^H]Serotonin uptake on platelets was used as marker of the functionality of SERT. *V*_max _and *K*_m _values of [^3^H]serotonin uptake in patients with FM accounted for 90 ± 4 pmol/10^9 ^platelets per minute and 96 ± 15 nM, respectively (means ± SEM; Table 3). A comparison of these parameters with those in healthy volunteers indicated significant differences for *V*_max _values but not for *K*_m _values: whereas the mean *V*_max _in the patients with FM was significantly lower (90 ± 4 versus 114 ± 6 pmol/10^9 ^platelets per minute for controls; *p *< 0.001), the mean *K*_m _in the patients with FM was not significantly different (96 ± 15 versus 114 ± 15 nM for controls; Table 2).

The severity of the symptoms was related to *V*_max _and *K*_m _in that the statistical analysis showed a negative correlation between the symptom scores and the respective *V*_max _values (*p *< 0.0001 for tiredness; *p *< 0.05 for TPi and *p *< 0.001 for FIQ). We also found a positive correlation between *K*_m _values and tiredness (*p *< 0.001), TPi (*p *< 0.001) and FIQ (*p *< 0.001).

The covariate age was shown not to have an effect on the variability of the *V*_max _or *K*_m _values in either patients with FM or healthy volunteers.

## Discussion

The present study is the first examination of the relationships between SERT expression and kinetic parameters of 5-HT uptake. We have found statistically significant differences in *B*_max _and *V*_max _values between patients with FM and controls. Moreover, we found a negative correlation between symptom scores and the respective *B*_max _and *V*_max _values (*B*_max _and *V*_max _versus tiredness, TPi and FIQ score). The platelet is considered to be a peripheral model of neuronal activity with respect to 5-HT function. In fact, previous studies have demonstrated that the same SERT is expressed in the central nervous system and platelets [14]. Moreover, the identity between the two structures, as confirmed by sequence homologies through cloning studies [15], has provoked a surge of different studies in neuropsychiatric disorders, given the possibility of exploring peripherally a mechanism of the central nervous system [26]. Recently it has been proposed that altered serotonergic neuronal function might be related to the pathophysiology of FM [5,8,27]. These findings prompted us to investigate the characteristics of SERT in the platelets of patients with FM. *B*_max _and *K*_d _values of [^3^H]Paroxetine binding were assumed to represent SERT density and ligand binding affinity, respectively, whereas *V*_max _and *K*_m _values of [^3^H]Serotonin uptake were taken as estimates of SERT rate and affinity, respectively. A very interesting observation was that both binding and uptake parameters differed significantly from those of healthy volunteers.

The patients with FM have fewer SERTs expressed on the cellular membrane than healthy subjects (a decreased *B*_max_, perhaps because the SERTs are less transcribed). Besides having fewer SERTs, patients with FM have a deficit in functionality (demonstrated by a decrease in transport rate).

Such combined changes in *B*_max _and *V*_max _values allow the inference that the efficiency of 5-HT uptake by platelet SERT is altered. Our previous studies demonstrated an alteration of SERT density and of the uptake rate of SERT in psychiatric patients [28,29]. Consistent with this suggestion was the correlation analysis in the present study: the lower the density and rate of SERT on platelet membranes, the higher the severity of FM symptoms. Moreover, the *K*_m _values were also positively correlated with tiredness, TPi and FIQ.

A reduced density and rate of SERT are consistent with previous observations indicating that levels of 5-HT are altered in patients with FM [30,31]. The biophysiological mechanism of FM has been proposed to be similar to that in depression, and it has been suggested that this is likely to result from a neuroendocrine/neurotransmitter dysregulation [32]. However, we suppose that the alterations in *B*_max _and *V*_max _values are not related to the pathophysiology of FM but are a consequence of FM. Our hypothesis is that a decrease in *B*_max _and *V*_max _of SERT is due to a pain stimulus [33,34].

It has been shown that the decreased pain perception threshold during depression is likely to result from a dysfunction in several neurotransmitter systems, especially the serotoninergic one, which is also involved in the pathophysiology of depression [35]. In addition, an excessive stimulation of peripheral 5-HT receptors would account for pain and might explain why the clinical use of 5-HT_3 _receptor antagonists such as tropisetron or granisetron can promote the relief of disturbance associated with FM [11,36].

SERT has been investigated previously in patients with FM, with discordant results. Russell and colleagues [37] found a higher *B*_max _in patients with FM than in healthy controls, whereas other authors found normal *B*_max _values [28,35] using [^3^H]Paroxetine or [^3^H]Imipramine [38]. In our experiments we used [^3^H]Paroxetine, which binds with high affinity to a specific population of binding sites located on human platelets and neuronal membranes, associated with 5-HT uptake mechanisms [39]. The present results indicate a decrease in the density and rate of platelet SERT in patients with FM, and allow us to propose a specific role for SERT in the pathogenesis of FM. In fact, we avoided the inclusion of patients with FM who had psychiatric components because it is known that in psychiatric disorders such as depression, the expression of SERT is altered [40-42] and it is very difficult to identify the role of the two components in patients with FM who have comorbid psychiatric disorders. Thus, the changes in *B*_max _and *V*_max _demonstrated in our study may be due to FM only.

There is also a possible contribution from 5-HT to the aetiology of FM because of the efficacy of SSRIs in the management of chronic pain in idiopathic pain disorders [43]. Thus, in view of the decreased *B*_max _and *V*_max _values found in our subset of mentally healthy patients, who were SSRI free, we propose that there is a compensatory mechanism in the central nervous system to relieve the pain. This may clarify the improvement in the therapeutic effectiveness of SSRI in the patients with FM.

## Conclusion

This is to our knowledge the first observation that, apart from a decrease in expression, there is also an alteration in the rate of SERT which seems to depend not only on the SERT number. In fact, *B*_max _is not correlated with *V*_max _(data not shown).

In the patients with FM, the decrease in 5-HT levels, which had already been observed, together with the impaired SERT functionality, might contribute to the pathogenesis of the disease, both in quantity and rate. In fact, these two factors are important because they are correlated with the level of disease severity.

Thus, the results of the present study are in agreement with the hypothesis that a deficit in the 5-HT transporter site could be of pathogenetic significance in FM syndrome.

## Abbreviations

5-HT = serotonin; *B*_max _= maximal membrane binding capacity; FIQ = Fibromyalgia Impact Questionnaire; FM = fibromyalgia; *K*_d _= dissociation constant; *K*_m _= Michaelis constant; SERT = specific serotonin transporter; SSRI = selective 5-HT reuptake inhibitor; TPi = tender point index; *V*_max _= maximal uptake rate of SERT.

## Competing interests

The authors declare that they have no competing interests.

## Authors' contributions

L Bazzichi was responsible for the design of the study and patient recruitment. GG was responsible for the design of the study and was the supervisor of the laboratory analysis. LB drafted the manuscript and was the coordinator of the laboratory activity. GM performed the statistical analysis. LF and PI conducted the binding assays. FDF and TG were responsible for the uptake assays. CG drafted the manuscript and performed the statistical analysis. AR was responsible for the preparation of the samples and for their storage. AL and SB were general supervisors of the research group. All authors read and approved the final manuscript.

## Figures and Tables

**Table 1:** [^3^H]Paroxetine binding characteristics in platelet membranes of control subjects and patients with fibromyalgia

Variable	Controls (*n *= 38)	Patients with FM (*n *= 40)
*B*_max _(fmol/mg protein)	1,260 ± 34	1,048 ± 30^a^
*K*_d _(nM)	0.077 ± 0.012	0.086 ± 0.021

Results are shown as means ± SEM. *B*_max_, maximal membrane binding capacity; FM, fibromyalgia; *K*_d_, dissociation constant. ^a^*p *< 0.0001.

**Table 2:** Characteristics of control subjects and patients with fibromyalgia

Characteristic	Healthy controls	Patients with FM	Reference range
Age (years)	50 ± 12	53 ± 13	-
Sex	F	F	-
Tender points	2 ± 0.5	14.3 ± 3.72	0–18
TPi	1 ± 0.5	2.2 ± 0.5	0–3
FIQ	22.3 ± 16.33	57.1 ± 21.3	0–100
Tiredness	2.73 ± 2.7	7.4 ± 2.2	0–10

Results are means ± SD. FIQ, Fibromyalgia Impact Questionnaire; FM, fibromyalgia; TPi, tender point index.

**Table 3:** Serotonin uptake characteristics in platelets of control subjects and patients with fibromyalgia

Variable	Controls (*n *= 38)	Patients with FM (*n *= 40)
*V*_max _(pmol/10^9 ^platelets per minute)	114 ± 6	90 ± 4^a^
*K*_m _(nM)	114 ± 15	96 ± 15

Results are shown as means ± SEM. FM, fibromyalgia; *K*_m_, Michaelis constant; *V*_max_, maximal uptake rate of specific serotonin transporter. ^a^*p *< 0.001.
